# Patient-Tailored Contrast Medium Volume for Preoperative Computed Tomography Angiography of the Aorta Based on Patient’s Heart Rate and Body Surface Area

**DOI:** 10.5334/jbsr.4033

**Published:** 2025-12-05

**Authors:** Miloud Dewilde, Walter Coudyzer, Annouschka Laenen, Hilde Bosmans, Geert Maleux

**Affiliations:** 1 University Hospitals KU Leuven, Leuven, Belgium; 2 Leuven Biostatistics and Statistical Bioinformatics Centre, Leuven, Belgium

**Keywords:** CT aortography, contrast medium

## Abstract

*Objectives:* To evaluate the impact of patient-specific contrast volume adjustments on preoperative aortic computed tomography angiography (CTA) image quality after adjusting and reducing the total, injected contrast volume based on patients’ body surface area (BSA) and heart rate (HR) and after adapting the CT-scanner’s kilovoltage (kV).

*Methods:* Prospective study included 80 surgery-naive patients. Three study groups were included: Group 1 (*n* = 56 patients): the injected contrast dose, calculated from BSA and HR, was reduced by 50%; Group 2 (*n* = 11 patients): the injected contrast dose, calculated from BSA and HR, was reduced by 50%, and an additional volume reduction was based on the kV values; Group 3 (*n* = 13 patients): the injected contrast dose, calculated from BSA and HR, was reduced by 50% and additionally diluted to 80% contrast and 20% saline. Image quality was evaluated by quantitative analysis (Hounsfield units) and qualitative analysis (five-point visual score).

*Results:* The mean injected contrast dose was 46.1 ml, 28.3 ml, and 35.0 ml in Groups 1, 2, and 3, respectively, with a significant difference between Group 1 vs Group 2 (*P* < 0.001) and between Group 1 vs Group 3 (*P* < 0.001). A linear relationship between the Hounsfield units and the given contrast dose for all study groups was observed. The mean image quality score for Group 1 was 4.34/5. The mean image quality score for Group 2 was 2.8/5 and for Group 3 was 3.5/5.

*Conclusions:* Significant contrast dose reduction, based on HR and BSA, in preoperative aortic CTA is associated with acceptable diagnostic quality.

## Introduction

Computed tomography angiography (CTA) of the aorta is considered as an important imaging modality for the identification and monitoring of pathologies affecting the thoraco-abdominal aorta. These pathologies encompass aneurysmal dilation, dissection, penetrating ulcer, aortic wall inflammation, luminal stenosis, and thrombo-embolic diseases [1]. Many health-care facilities use standardized CTA scanning protocols with fixed doses of nonionic contrast medium. Traditionally, a standard dose of 120 milliliters (ml) of iodinated contrast medium has been injected, and this procedure was maintained despite advancements in CT technology and image reconstruction techniques, including modifications in tube voltage, enhanced image acquisition speed, and the use of multiphasic contrast injection strategies. More recently, several researchers have advocated the adoption of lower contrast doses while maintaining diagnostic efficiency of the CTA imaging [2–9].

Furthermore, investigators have elucidated that contrast enhancement is significantly determined by patient’s cardiopulmonary and habitus status, in particular patient’s body surface area (BSA) and heart rate (HR) [10–15]. Consequently, a pragmatic calculator was developed to determine the optimal contrast dose based on patient’s HR and BSA. In a pilot study by Raymakers et al., a contrast agent dose calculator was used for CTA of the aorta [16]. Image quality appropriateness was assessed through a quantitative and qualitative evaluation comparing CTA images obtained after injection of a reduced volume of contrast medium versus a fixed contrast agent dose of 120 ml. Subsequently, in a second phase, the feasibility of further diluting the calculated volumes by 50% was explored showing that contrast doses in CTA could be substantially decreased without compromising diagnostic efficacy.

Our study started from this successful low contrast agent dose with its 50% reduction. Two techniques were used next, to test whether these contrast volumes could even be lowered. One approach lowered the contrast dose based on the kilovoltage (kV) of the CT scan. Alternatively, the contrast volume was reduced by diluting the contrast volumes to 80%.

## Materials and Methods

### Study design and inclusion and exclusion criteria

This is a prospective, single-center study involving patients undergoing CTA of the thoraco-abdominal or abdominal aorta for the detection or follow-up of aortic diseases. Approval for this study was obtained from the institutional Ethics Committee (S58042), and patients provided informed consent before undergoing the CTA. From March 2020 till December 2023, the included patients were assigned to one of three study groups and underwent scanning according to a standardized protocol. The first cohort of 56 included patients were assigned to Group 1; the next cohort of 11 included patients to Group 2, and the last cohort of 13 patients were assigned to Group 3. Contrast dose calculations were facilitated by an injection calculator (iCalc, Medicor International, Rotselaar, Belgium), integrated into the contrast dose injector on-site, allowing for seamless input by CT technicians prior to scanning.

CTA was conducted for diagnostic or follow-up imaging investigation related to abdominal aorto-iliac and visceral aneurysms, dissections, or aortic occlusive disease. Patients with a history of previous aortic surgery, including endovascular aortic repair (EVAR) or stenting, were excluded from the study. All measurements and evaluations were focused on the aorta in thoraco-abdominal and abdominal CT scans. Isolated thoracic CTA aortography cases were not included in the study.

Patients under follow-up for cardiac disorders were also excluded from the study. Finally, individuals with contraindications for contrast administration (e.g., significant adverse reactions or creatinine levels exceeding 2.0 mg/dl) and those lacking intravenous access suitable for a bolus contrast injection of 3 cc/s were also excluded from participation.

### Contrast agent dose calculation

Three study groups were created: Group 1 (iCalc value −50%) received a contrast dose dependent on their BSA and HR calculated by the iCalc injection calculator minus 50%; Group 2 (iCalc value −50% + extra reduction) received 50% of the injector-calculated contrast agent dose with extra reduction on the basis of scan parameters (kV) supplemented with saline to the same volume as before reduction; and Group 3 (iCalc −50% + extra dilution) received 50% of the injector-calculated contrast agent dose with extra dilution in a proportion of 80% contrast and 20% saline.

The formula underpinning the calculator incorporates four identical components, as described earlier. First, BSA was computed using the formula: ((length (cm) × weight (kg))/3600) × 0.5. Subsequently, BSA was multiplied by a fixed contrast dose of 45 ml/m^2^. This determination was informed by the observations of a Japanese research group [14], which indicated that aortic enhancement remained consistent when employing a protocol administering a fixed value of 42.51 ml/m^2^. We selected a slightly higher value of 45 ml/m^2^ arbitrarily.

This data was entered into the calculator by the radiographer. Contrast agent injection was carried out at a standard rate of 3 cc/s and followed by a 25 ml saline flush at the same rate.

### Scanning protocol

All acquisitions were conducted with a 192-detector-row CT scanner (Siemens Somatom Force, Erlangen, Germany). A single-energy mode was employed for all scans in the study. The imaging parameters were as follows: detector collimation of 192 × 0.6 mm, helical pitch of 0.6, gantry rotation time of 0.5 s, tube voltage of 100 kV for patients of normal size, and a planned tube current–time product per rotation of 90 mAs for patients of normal size. Automatic tube current modulation, facilitated by the Care dose 4D and Care kV dose reduction options, allowed us to adjust tube loads according to varying patient sizes.

As per clinical requirements, imaging procedures were conducted with varying contrast administration protocols, encompassing scans conducted without contrast, during the arterial phase and/or during the venous phase. An arterial phase scan was consistently performed for all subjects, and this scan was analyzed in present study.

Patients diagnosed with an aneurysm underwent both native and arterial phase CTA scans. Conversely, patients presenting with a dissection underwent an arterial phase CT scan followed by a subsequent late, venous phase CT scan. The determination of scan delay was personalized utilizing a bolus-tracking technique. The injection rate remained uniform across all patients and was fixed at 3 cc/s. Triggering of the imaging acquisition was executed upon attainment of intra-aortic enhancement reaching a threshold of 120 Hounsfield units (HU) at the level of the diaphragmatic region.

In patients presenting with thoraco-abdominal pathology, the field of view encompassed from the clavicles to the bilateral inguinal region, whereas in cases of abdominal aortic pathology, it extended from the diaphragms to the bilateral inguinal region. The slice thickness employed for native images was 1 mm with a slice increment of 0.7 mm. Axial reformation offered reconstructed images at both 3 and 1 mm thicknesses, while coronal reformation was made accessible at a thickness of 3 mm for visualization within the Picture Archiving and Communication System (Enterprise, Agfa Gevaert, Mortsel, Belgium) platform.

The injected iodinated contrast agents encompassed Iomeprol 350 (Iomeron, Bracco, 350 mg I/ml), Iobitridol 350 (Xenetix, Guerbet, 350 mg I/ml), Iodixanol 320 (Visipaque, GE Healthcare, 320 mg I/ml), and Iopromide 370 (Ultravist, Bayer, 370 mg I/ml). The stock concentration of the contrast medium was adjusted within the injector calculator and duly documented in the dataset. Contrast dosage was administered in varying protocols: either 50% of the calculated value from the injector calculator (Group 1), 50% of the calculated value from the injector calculator with an additional contrast dose reduction based on kV (Group 2), or 50% of the calculated value from the injector calculator with an 80/20 dilution (Group 3). Finally, a standardized volume of saline (25 cc) was utilized for contrast flushing.

### Quantitative image analysis

Quantitative analysis entailed the HU measurement at predetermined anatomical levels within the abdominal aorta, including the suprarenal level (coeliac trunk), renal level, infrarenal level (mid-aorta), and midway level through both common iliac arteries. A circular region of interest (ROI) was meticulously positioned at the center of the aorta or common iliac arteries or, in instances of dissection, centrally within the true lumen. To facilitate comparison across patients, the mean HU value across all five levels was calculated. Furthermore, the lowest and highest HU values recorded at any level were scrutinized, and the corresponding images were selected for a comparative analysis.

### Qualitative image analysis

Two radiologists with 5 and 30 years of experience in vascular radiology respectively used visual grading analysis to perform a qualitative assessment. Readings were done independently to allow interobserver comparison. Visual scoring was based on a five-point visual scale and earlier used in other studies; briefly, score 1 = inadequate/nondiagnostic; score 2 = suboptimal/barely diagnostic; score 3 = sufficient/diagnostic; score 4 = good; score 5 = excellent. A score of 3 or more was considered clinically acceptable, whereas a score of 2 was suboptimal and 1 was unacceptable for diagnostic purposes.

### Interobserver image analysis

Agreement was quantified by (1) the proportion of absolute agreement and (2) the weighted Kappa coefficient of agreement: Kappa ≤ 0 indicating no agreement and 0.01–0.20 as none to slight, 0.21–0.40 as fair, 0.41–0.60 as moderate, 0.61–0.80 as substantial, and 0.81–1.00 as almost perfect agreement.

### Statistical analysis

Analyses have been performed using SAS software (version 9.4 of the SAS System for Windows, Cary, NY, USA). To qualify the agreement between the two raters (interobserver agreement), the absolute agreement proportion was estimated with 95% confidence interval as well as the weighted Kappa coefficient of agreement. The average score of both raters was obtained and further used as the qualitative score. The association between the qualitative and quantitative score and between the qualitative and given contrast dose was estimated by the Spearman correlation coefficient. The Kruskal–Wallis test was used for group differences of the total contrast dose, and pairwise comparisons were performed by the Mann–Whitney *U* test. Patient parameters (age, weight, height, body mass index, and HR) are presented as mean, median, standard deviation, interquartile range, and range (minimum–maximum).

## Results

### Patient enrollment and patients’ characteristics

A total of 92 patients were initially enrolled and included in one of the three study groups. A total of 91 CTA scans were performed; one patient finally did not undergo a CT scan and was excluded from the study. Another eleven patients did not receive their scan according to the predetermined scan protocol and were also excluded from the study. Finally, a total of 80 patients (23 females, 57 males, median age 71 years, and range 41–90 years) were included in the study and received an aortic CTA scan according to the study group. Patient characteristics are summarized in Table 1.

**Table 1:** Patient demographics.

DEMOGRAPHIC PARAMETER	GROUP 1 (*N* = 56)	GROUP 2 (*N* = 11)	GROUP 3 (*N* = 13)	TOTAL (*N* = 80)
Sex: men	44 (79%)	6 (55%)	7 (54%)	57 (71%)
Age	71 (std 11.2)	70 (std 11.7)	64 (std 12.1)	69.6 (std 69.6)
Weight (kg)	78.1 (std 15.1)	78.3 (std 15.6)	71.9 (std 17.4)	77.1 (std 15.5)
Height (cm)	173 (std 7.9)	166 (std 9.2)	171 (std 6.7)	171 (std 8.1)
HR (bmp)	73 (std 14.6)	75 (std 11.1)	74 (std 15.4)	73 (std 14.2)
Indication for CTA:				
Infrarenal AAA	23	5	3	31
Iuxtarenal AAA		1	1	
TAAA	16	3		19
TBAD	7	1	2	10
TAAD	5		3	8
Aortic stenosis	2	1	1	4
PAU	1			1
IAID	1			1
CIAA	1	1	1	3
Celiac trunk dissection			1	1
Renal artery aneurysm			1	1

kg: kilogram

cm: centimeter

HR: heart rate

bmp: beats per minute

AAA: abdominal aortic aneurysm

TAAA: thoraco-abdominal aortic aneurysm

TBAD: type-B aortic dissection

TAAD: type A aortic dissection

PAU: penetrating aortic ulcer

IAID: iatrogenic aorti-iliac dissection

CIAA: common iliac artery aneurysm

Per protocol, *n* = 56 patients were assigned to Group 1, *n* = 11 patients to Group 2, and *n* = 13 patients to Group 3.

The upper limit of 60 cc of contrast was given in *n* = 5 patients, all in Group 1. In three patients, a 3 cc/s injection rate could not be given, and therefore 2.5 cc/s was chosen.

### Contrast agent dose calculation

**Figure 1:**
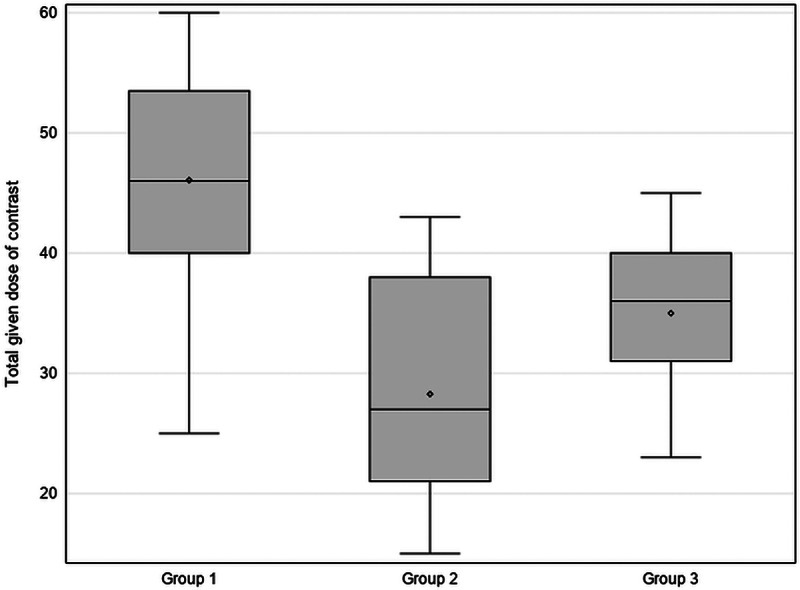
Box plot of mean total injected contrast dose per study group. A significant difference between Groups 1 and 2 (*P* < 0.001) and between Groups 1 and 3 (*P* < 0.001) is found. No clear difference in between the injected contrast dose between Groups 2 and 3 is demonstrated (*P* = 0.072).

**Figure 2 (a, b):**
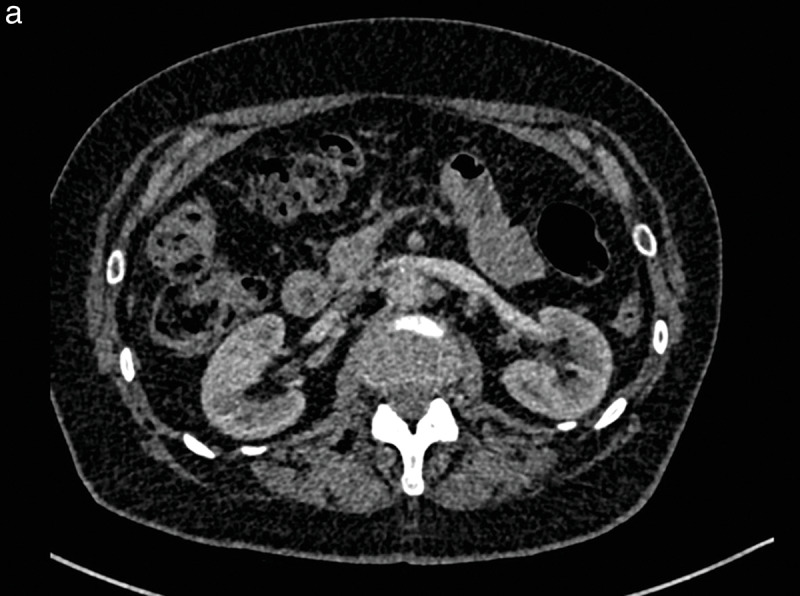

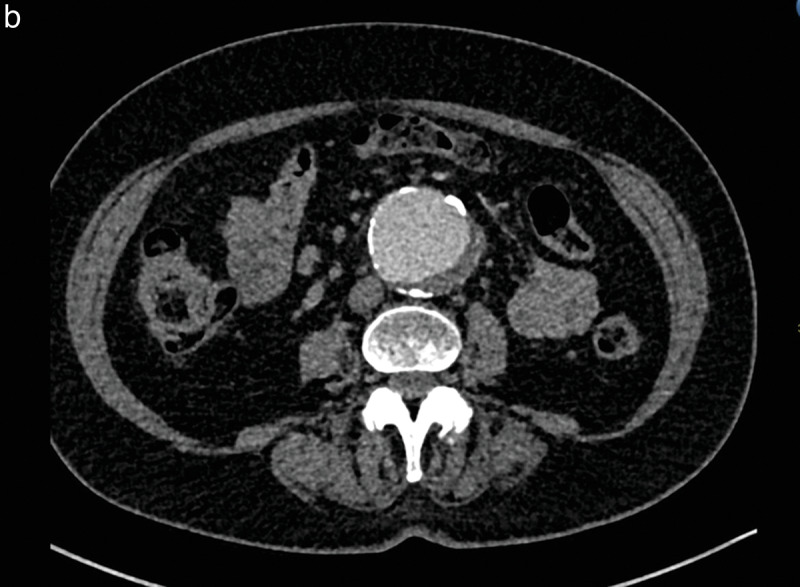
Patient with an abdominal aortic aneurysm, included in study Group 2. After intravenous injection of 15 cc of iodinated contrast medium, the qualitative imaging score was 2 by the two independent observers for the axial slice at the renal artery level **(a)** and was 3 at the infrarenal level **(b)**.

Contrast doses for the three study groups are summarized in Table 2. The mean contrast dose was 46.1 ml, 28.3 ml, and 35 ml in Group 1, Group 2, and Group 3, respectively, with a significant difference between Group 1 vs Group 2 (*P* < 0.001) and between Group 1 vs Group 3 (*P* < 0.001). No significant difference was found between Groups 2 and 3 (*P* = 0.072) (Figure 1). In *n* = 5 patients in Group 1, calculated doses were higher than 120 ml due to a combination of high body weight, large stature, and/or high HR; however, the maximum amount of injected contrast medium was 60 ml per protocol; the maximum injected contrast volume was 43 and 45 ml in Groups 2 and 3, respectively. The lowest contrast dose in this study was 15 ml injected in one patient with an abdominal aortic aneurysm (Group 2), with enhancement near to 177 HU of the abdominal aneurysm, but significantly lower at the other four points of visual scoring (lowest 109 HU) (Figure 2 a and b) and a low qualitative visual score of 2/5.

**Table 2:** Calculated doses of injected contrast medium.

CONTRAST DOSE	GROUP 1	GROUP 2	GROUP 3	TOTAL
iCalc (ml)	92 (std 18)	93 (std 21)	89 (16)	92 (std 18)
Total injected dose	46 (std 9)	28 (std 10)	35 (std 6)	42 (std 11)

ml: milliliter

### Quantitative image analysis

Mean enhancement for the three groups is summarized in Table 3. Overall, there is a linear relationship between the HU and the administered contrast dose for Groups 1 and 2 but not in Group 3 as summarized in Table 4. Furthermore, there is also a clear proportional relationship between the amount of administered contrast dose and the quantitative score for Group 1 (*P* < 0.0001), Group 2 (*P* = 0.004), and Group 3 (*P* = 0.05), respectively, as summarized in Table 5.

**Table 3:** Quantitative image analysis of mean pixel values (in Hounsfield units) from ROIs in different anatomical regions.

ANATOMIC SEGMENT OF THE AORTA	GROUP 1	GROUP 2	GROUP 3	TOTAL
Suprarenal aorta	345 (std 101)	191 (std 83)	268 (std 87)	311 (std 110)
Renal aorta	342 (std 101)	193 (std 78)	263 (std 81)	309 (std 109)
Infrarenal aorta	350 (std 100)	203 (std 83)	254 (std 80)	314 (std 109)
Left common iliac artery	352 (std 98)	197 (std 76)	248 (std 81)	316 (std 110)
Right common iliac artery	342 (std 96)	195 (std 85)	237 (std 85)	306 (std 108)

**Table 4:** Linear correlation coefficient between the total administered dose and Hounsfield units measured at different levels of the aorto-iliac region.

ANATOMIC REGION	RHO	95% CI	*P*-VALUE
**GROUP 1 (56 PATIENTS)**			
Suprarenal aorta	0.353	(0.096; 0.561)	0.0073
Renal aorta	0.424	(0.178; 0.616)	0.0010
Infrarenal aorta	0.354	(0.097; 0.562)	0.0071
Left CIA	0.218	(−0.049; 0.454)	0.1060
Right CIA	0.217	(−0.051; 0.452)	0.1090
**GROUP 2 (11 PATIENTS)**			
Suprarenal aorta	0.765	(0.271; 0.931)	0.0043
Renal aorta	0.729	(0.195; 0.919)	0.0088
Infrarenal aorta	0.706	(0.150; 0.912)	0.0129
Left CIA	0.699	(0.086; 0.917)	0.0220
Right CIA	0.711	(0.109; 0.920)	0.0186
**GROUP 3 (13 PATIENTS)**			
Suprarenal aorta	0.493	(−0.100; 0.814)	0.0876
Renal aorta	0.433	(−0.173; 0.787)	0.1432
Infrarenal aorta	0.342	(−0.271; 0.745)	0.2604
Left CIA	0.460	(−0.141; 0.800)	0.1158
Right CIA	0.521	(−0.064; 0.826)	0.0679

CI: confidence interval

**Table 5:** Qualitative images scores.

VISUAL SCORE	GROUP 1	GROUP 2	GROUP 3	TOTAL
**INVESTIGATOR 1**				
1	1/56 (1.8%)	4/11 (36.4%)	1/13 (7.7%)	6/80 (7.5%)
2	3/56 (5.4%)	0/11 (0%)	3/13 (23.1%)	6/80 (7.5%)
3	5/56 (8.9%)	4/11 (36.4%)	0/13 (0%)	9/80 (11.2%)
4	17/56 (30.4%)	3/11 (27.3%)	6/13 (46.1%)	26/80 (32.5%)
5	30/56 (53.6%)	0/11 (0%)	3/13 (23.1%)	33/80 (41.2%)
**INVESTIGATOR 2**				
1	1/56 (1.8%)	2/11 (18.2%)	2/13 (15.4%)	5/80 (6.2%)
2	1/56 (1.8%)	2/11 (18.2%)	2/13 (15.4%)	5/80 (6.2%)
3	3/56 (9.1%)	1/11 (9.1%)	0/13 (0%)	4/80 (5.0%)
4	21/56 (37.5%)	5/11 (45.4%)	7/13 (53.8%)	33/80 (41.2%)
5	30/56 (53.6%)	1/11 (9.1%)	2/13 (15.4%)	33/80 (41.2%)

### Qualitative image analysis

**Figure 3:**
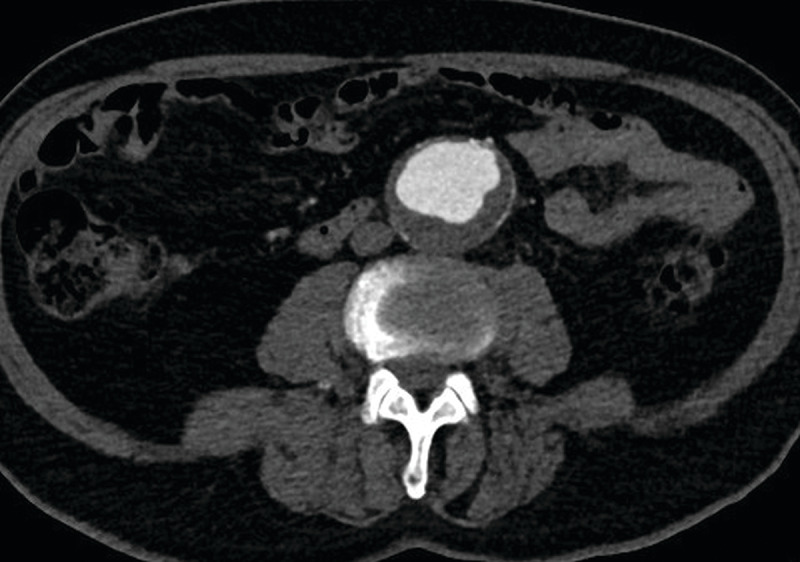
Patient with an infrarenal aortic aneurysm, included in study Group 3. After intravenous injection of 38 cc of iodinated contrast medium (with 9 cc physiologic saline), the qualitative score was 5 by the two independent observers. The quantitative score was 372 Hounsfield units at the level of the abdominal aortic aneurysm.

Image quality scores are summarized in Table 5. Mean image quality for Group 1 was scored 4.34/5. There was one patient with a score of 1 and two patients with a score of 2. The majority of patients in Group 1 had a score of 4 or 5. Mean image quality for Group 2 was scored 2.82/5 and for Group 3 was scored 3.46/5 (Figure 3). In Group 2, there were *n* = 4 patients with a score of 1 or 2. In Group 3, there were also *n* = 4 patients with a score of 1 or 2. But no patients needed to be recalled for repeat imaging related to image quality that was judged insufficient for correct diagnosis. Furthermore, there is also a clear proportional relationship between the amount of administered contrast dose and the qualitative score for Group 1 (*P* < 0.0001), Group 2 (*P* = 0.004), and Group 3 (*P* = 0.05), respectively, as summarized in Table 6.

**Table 6:** Relationship between the total amount of administered contrast dose and the qualitative scores adjudicated at different levels of the aorto-iliac region.

ANATOMIC REGION	RHO	95% CI	*P*-VALUE
**GROUP 1 (56 PATIENTS)**			
Suprarenal aorta	0.818	(0.703; 0.888)	<0.0001
Renal aorta	0.859	(0.766; 0.914)	<0.0001
Infrarenal aorta	0.815	(0.699; 0.886)	<0.0001
Left CIA	0.717	(0.554; 0.822)	<0.0001
Right CIA	0.750	(0.602; 0.844)	<0.0001
**GROUP 2 (11 PATIENTS)**			
Suprarenal aorta	0.924	(0.704; 0.979)	<0.0001
Renal aorta	0.942	(0.769; 0.984)	<0.0001
Infrarenal aorta	0.929	(0.720; 0.980)	<0.0001
Left CIA	0.988	(0.942; 0.997)	<0.0001
Right CIA	0.988	(0.942; 0.997)	<0.0001
**GROUP 3 (13 PATIENTS)**			
Suprarenal aorta	0.728	(0.268; 0.908)	<0.0035
Renal aorta	0.661	(0.146; 0.882)	<0.0120
Infrarenal aorta	0.725	(0.262; 0.907)	<0.0037
Left CIA	0.882	(0.623; 0.962)	<0.0001
Right CIA	0.780	(0.375; 0.927)	0.0009

CI: confidence interval

CIA: common iliac artery

Our Kappa values for interobserver agreement were moderate (Kappa = 0.577 and Kappa = 0.576) in Groups 1 and 3, respectively, and fair in Group 2 (Kappa = 0.282) (Table 7), with a consistent difference in scoring.

**Table 7:** Interobserver agreement analysis.

GROUP	PROPORTION OF AGREEMENT (95% CI)	WK COEFFICIENT	*P*-VALUE
Group 1	0.750 (0.622; 0.846)	0.577 (0.384; 0.770)	<0.001
Group 2	0.455 (0.213; 0.720)	0.282 (−0.059; 0.624)	0.0526
Group 3	0.692 (0.420; 0.876)	0.576 (0.238; 0.914)	0.0004

CI: confidence interval

WK coefficient: weighted Kappa coefficient, Kappa (95% CI)

## Discussion

This study demonstrates a robust diagnostic efficacy of CTA for abdominal aortic disorders with a mean contrast dose injection of 46 ml, based on an injector system determining contrast agent volume that was reduced to 50% (Group 1), which is in line with earlier findings by Raymakers et al. [16] revealing an equally high diagnostic abdominal CTA performance using a mean contrast dose of 48 ml. Compared to traditional injection volumes of 120 ml of iodinated contrast medium, up to 60% dose reduction can be obtained without decrease in qualitative imaging performance. In addition to the 50% contrast dose reduction based on iCalc calculation, this study also found diagnostically acceptable imaging quality when reducing the contrast volume even more, based on kV adaptation, down to 28 ml (Group 2). Chen et al. using a 320-row volume CT for abdominal CTA and a low tube voltage of 80 kVp found no diagnostic imaging difference in between abdominal CTA images obtained after intravenous injection of 100 ml of iodinated contrast medium and scanned at a standard tube voltage of 120 kVp, compared to intravenous injection of 40 ml of iodinated contrast medium and scanned at a low-dose tube voltage of 80 kVp [2]. Nakayama et al. also used a reduced tube voltage of 90 kVp and 40 ml of intravenous injection of iodinated contrast medium and found better image quality on 16-MDCT aortography in lighter patients (<70 kg) compared to heavier patients (>70 kg) [7]. In our study, we did not make a subanalysis with regard to patient’s body weight related to the limited number of patients included and the lack of statistical power.

Last, in this study an additional dilution of injected contrast dose with 20% of saline (Group 3) and resulting in a mean 35 ml of iodinated contrast medium injected was also associated with clinically acceptable CTA performance. However, image quality in Groups 2 and 3, based on a qualitative and quantitative evaluation, was clearly lower compared to image quality in Group 1; still the vast majority of the CT-imaging studies, and in particular the cases of Group 3, were of acceptable efficacy without the clinical need for repeat CTA that would have cost a higher dose of iodinated contrast medium. This increased contrast dose reduction method might be an interesting clinical imaging tool for patients with impaired kidney function and strict need for contrast-enhanced CTA or for patients with strict need for repetitive aortic CTA imaging follow-up. It also shows the under limit of current CTA scans of the abdominal aorta.

Finally, this study also has some limitations. First, the study includes an arbitrary designation of contrast volume per BSA to a static value of 45 ml/m^2^, irrespective of specific characteristics of the contrast agent or scan parameters; in addition, lean body mass might be a better parameter than BSA for vascular attenuation analysis as demonstrated by Henning et al. in chest CT [17]. Second, although this is a prospective study with three well-defined study group cohorts, patients were not randomized. Third, patients with renal or cardiac function impairment were excluded from the study, and the study conclusions might not be extrapolated to all, in particular to patients with left heart failure. Fourth, only aortic surgery-naive patients were included; and the study results cannot be extrapolated to aortic CTA in patients referred for imaging follow-up after open or endovascular aortic surgery, including (complex) EVAR procedures. Fifth, although a moderate to fair interobserver variability was found, no intraobserver analysis was performed. Sixth, various types of aortic diseases were scanned, including aneurysms and (chronic) dissections; however, it is still unclear if the contrast dose reduction would be equally effective for these different types of aortic diseases. Last, this study was performed using multidetector CT scanners without photon-counting technology. However, further investigations are warranted to comprehensively evaluate the implications of additional reduction and dilution of contrast, predicated on scanning parameters and predefined substantial dilutions, respectively.

## Conclusion

The reduction of contrast doses in aortic CTA, while maintaining diagnostic efficacy, is feasible through the utilization of a contrast injection algorithm that incorporates HR and BSA, coupled with the potential for additional contrast volume reduction by up to 50%.
